# Design and clinical implementation of an open-source bionic leg

**DOI:** 10.1038/s41551-020-00619-3

**Published:** 2020-10-05

**Authors:** Alejandro F. Azocar, Luke M. Mooney, Jean-François Duval, Ann M. Simon, Levi J. Hargrove, Elliott J. Rouse

**Affiliations:** 1 Department of Mechanical Engineering, University of Michigan, Ann Arbor, MI USA; 2 Robotics Institute, University of Michigan, Ann Arbor, MI USA; 3 Dephy, Inc., Maynard, MA USA; 4 Center for Bionic Medicine, Shirley Ryan AbilityLab, Chicago, IL USA; 5 Department of Physical Medicine and Rehabilitation, Northwestern University, Chicago, IL USA; 6 Department of Biomedical Engineering, Northwestern University, Evanston, IL USA

**Keywords:** Electrical and electronic engineering, Mechanical engineering, Medical research, Biomedical engineering

## Abstract

In individuals with lower-limb amputations, robotic prostheses can increase walking speed, and reduce energy use, the incidence of falls and the development of secondary complications. However, safe and reliable prosthetic-limb control strategies for robust ambulation in real-world settings remain out of reach, partly because control strategies have been tested with different robotic hardware in constrained laboratory settings. Here, we report the design and clinical implementation of an integrated robotic knee–ankle prosthesis that facilitates the real-world testing of its biomechanics and control strategies. The bionic leg is open source, it includes software for low-level control and for communication with control systems, and its hardware design is customizable, enabling reduction in its mass and cost, improvement in its ease of use and independent operation of the knee and ankle joints. We characterized the electromechanical and thermal performance of the bionic leg in benchtop testing, as well as its kinematics and kinetics in three individuals during walking on level ground, ramps and stairs. The open-source integrated-hardware solution and benchmark data that we provide should help with research and clinical testing of knee–ankle prostheses in real-world environments.

## Abstract (web summary)

An open-source integrated knee–ankle robotic prosthesis facilitates the testing of control strategies and biomechanics in real-world environments.

## Main

Millions of people with lower-limb amputations experience a reduced quality of life^1,2^. With the exception of highly athletic individuals, the majority of these people walk slower, get tired faster and are less stable compared with healthy individuals^3–6^. In the intact human body, leg muscles contract during walking to add mechanical energy^7^; however, traditional passive prostheses are not able to provide this energy and, subsequently, cannot restore the natural functions of muscles lost during an amputation. This renders more demanding activities, such as climbing stairs and ramps, particularly difficult^8^. Furthermore, individuals with lower-limb amputations often develop compensatory modifications to their gait, biomechanics and muscle activation patterns that lead to further complications, such as osteoarthritis, osteoporosis and back pain^9–11^. Finally, the resulting mobility challenges can lead to depression, social stigmatization and unemployment^2,12,13^. Although passive prostheses provide substantial mobility benefits, their physical, psychological and social impacts may limit the quality of life for many individuals with amputations.

Several research groups are developing powered knee^14,15^, ankle^16–19^ and knee–ankle (whole leg)^20–22^ prostheses that have the ability to produce able-bodied kinematics and kinetics that are not possible, or are extremely difficult, with passive systems^23–25^. These capabilities are typically achieved using electric motors that add net-positive mechanical energy analogous to the muscles within the leg. For example, powered knee prostheses have the ability to recreate early-stance knee flexion and extension—a region of the gait cycle that most passive prostheses cannot reproduce without an increased risk of falls^14,26,27^. Furthermore, powered ankle prostheses can add energy for powered push-off; by contrast, individuals with passive ankle prostheses often rely on exaggerated hip motions to compensate for the lack of push-off^10^. These powered knee–ankle systems combine the benefits of powered knee and ankle prostheses, and have the potential to further improve quality of life; however, they introduce the need for more complex, coordinated control strategies. As a consequence, results from studies of powered prostheses have often been debated. For example, although some individual experiments show patients walking faster, using less metabolic energy or exhibiting improved centre-of-pressure progression, there has been discussion about the applicability of these systems in clinical settings or for people with lower activity levels^28^. This is not necessarily a limitation of the prosthesis hardware but, rather, a lack of understanding about how best to control these devices. Finally, prosthesis emulators are a recently developed tool for quickly and systematically testing control systems^29–31^. Emulators utilize off-board motors and Bowden cable tethers, leading to high performance and low prosthesis weight; however, the tether between the motor and prosthesis can limit experiments to a laboratory setting with a treadmill.

There have also been promising advances in the development of safe, natural and intuitive control approaches. Today’s state-of-the-art control architectures typically include three overarching levels of control, each bearing responsibility for certain aspects of successful community ambulation. The control systems must recognize the user’s intended movement (that is, high-level control), translate the intended movement into an appropriate pattern of leg movement and effort (mid-level control) and execute the desired motions with closed-loop control (low-level control)^32^. Errors or failures at any of these levels may lead to falls, injuries, loss of confidence and reduced community mobility. Fortunately, this is an active area of research in which many groups are studying different approaches to the levels of control^33–38^. For example, recently developed high-level control approaches range from simple thresholds to machine learning techniques that automatically transition between control strategies for different ambulation modes, such as level-ground walking, ramp ascent/descent and stair ascent/descent^20,39^. Modern mid-level strategies implement impedance-based control, phase-based control or biologically-inspired neuromuscular models^20,33–35^. Finally, low-level control methods use a combination of feedback and feedforward control loops to minimize the error between the measured and desired states of the robot^32^. Overall, control systems for robotic legs are highly sophisticated, and most groups focus on a subset of high-level, mid-level or low-level control^32^. Although this practice avoids the challenges associated with integrating the three levels of control, it limits the impact of these controllers in real-world settings. Thus, despite promising research, key challenges remain in the development of control strategies that are safe, robust and intuitive.

Although talented researchers around the world are investigating the best ways to control robotic prostheses, the development of prosthetic hardware requires substantial investment of time and resources before research can begin. Even after research is complete, differences in design, performance and limitations hinder the ability to compare the merits of different control systems. For example, robotic prostheses today vary widely in size, weight, transmission type, controllability and degrees of freedom. Many research prostheses must also be tethered to a power supply, preventing researchers from testing in more challenging and realistic environments. Finally, as most research prostheses are prototypes, they are typically only tested in a few studies by the original designers, and can be difficult for other researchers to use. Commercially available emulator systems help to address many of these issues. Although these emulator systems are powerful, versatile and enable quick exploration of control systems, they do not have the portability or potential clinical relevance of a self-contained prosthetic leg. The lack of low-cost, high-performance and accessible powered prosthetic leg technology has hindered progress in the field and, ultimately, the quality of life of individuals with lower-limb amputations.

**Fig. 1:**
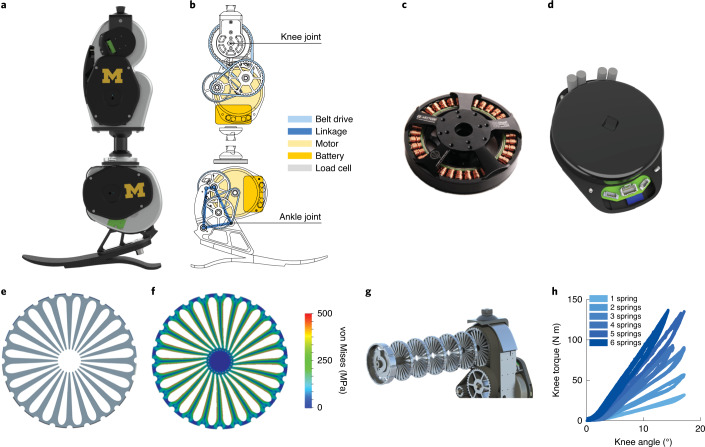
The OSL and its design components. **a**, Rendering of the OSL. **b**, Schematic of the OSL, highlighting the transmission, electronics and load cell. **c**, Output view of the electric motor used in the OSL. **d**, Output view of the motor integrated with the open-source motor controller and embedded system. **e**, Side view of a single spring disk. **f**, Finite element analysis of a spring disk being deflected by the gear-shaped internal shaft. The colours represent the von Mises stress. **g**, Exploded view of six springs stacked inside the knee output pulley. **h**, Torque-angle relationship of the knee with 1–6 springs stacked inside. Each spring has a stiffness of approximately 100 N m rad^−1^. We tested each condition five times.

To facilitate the study and fair comparison of control approaches, lower the barrier to performing controls research and prevent duplication of effort, we have created the Open Source Leg (OSL): a unique robotic knee–ankle prosthesis system developed for open-source adoption (Fig. 1 and Supplementary Video 1). The OSL includes prosthesis hardware, actuation, sensing, low-level control software and software libraries to communicate with researcher-specific mid- and high-level control systems. Here we present the design, technical characteristics and performance of the OSL. We provide a detailed description of the design process, mechatronics implementation and characterize multiple control architectures. We also highlight two design components—integration of high-torque motors and a customizable series-elastic actuator. Finally, we demonstrate patients walking with the OSL across level ground, ramps and stairs in a hospital setting, with all control parameters provided as reference for future researchers (Supplementary Video 1).

## Results

In addition to this Article, we have developed a companion website (www.opensourceleg.com) with detailed step-by-step guides to order parts, videos to build and test the hardware (Supplementary Video 1) and code to begin walking with the OSL using a preliminary control system (further details are provided in the Discussion).

To ensure that the OSL is accessible for researchers from a diverse range of backgrounds (such as controls, biomechanics and clinical), we abided by the following design principles:

1. Simple: the OSL can be assembled, controlled and maintained with moderate ‘hands-on’ skills. To this end, we reduced the number of components and suppliers; the vast majority of parts are machined from a single supplier, without dependencies on other precision machine components or mechanisms.
2. Portable: the OSL weighs less than the biological counterpart, and each joint has on-board batteries, sensing and control, facilitating research outside of the laboratory.
3. Scalable: the knee and ankle joints can operate independently, enabling research in patients with above-knee and below-knee amputations.
4. Customizable: the OSL includes several design and control features that can be customized depending on the specific requirements of the researcher, including the knee’s series elastic element, foot type and inclusion of a load cell, among other options.
5. Economical: the OSL costs approximately US$10,000–30,000 in prototype quantities, depending on degrees of freedom and sensing options, given the present manufacturing and material costs. By contrast, commercially available powered prostheses—such as the Ottobock emPOWER ankle and the Ossur POWER KNEE—cost up to US$100,000 each, without access to control modifications.

The intent of our design was to provide the highest performance, while facilitating ease of use, as well as reducing mass and cost. We implemented brushless electric motors from the drone industry because their efficiency and torque density permitted lower transmission ratios, enabling the use of timing belt drive transmissions instead of more expensive or complex alternatives that can have substantial product lead times and cost (such as harmonic drives and roller screw transmissions; Fig. 1). Furthermore, the OSL takes advantage of an open-source motor controller and embedded system, enabling researchers to focus on mid- and high-level control strategies, rather than developing low-level controllers and communication protocols. The OSL actuators have built-in position, current and impedance controllers, along with an inertial measurement unit (IMU) and a motor encoder. Together with Python and MATLAB interfaces, the actuators enable researchers to quickly begin control investigations with the OSL.

Portability and scalability are important characteristics that enable the OSL to be tested in various environments and by users with different levels of amputation. The low mass and compact power supply of the OSL enable research beyond typical lab-based treadmill tests. The OSL is shorter than the 4th percentile and lighter than the 16th percentile male shank and foot^7,40^. The housings completely encompass the transmissions, batteries and most of the electronics, reducing the risk of contamination or injury. Finally, the knee and ankle have independent embedded control hardware and batteries, enabling researchers to work with either the entire leg or a single joint. The portability and scalability of the OSL provide the ability to investigate control strategies in patients with above-knee and below-knee amputations in indoor and outdoor environments.

**Fig. 2:**
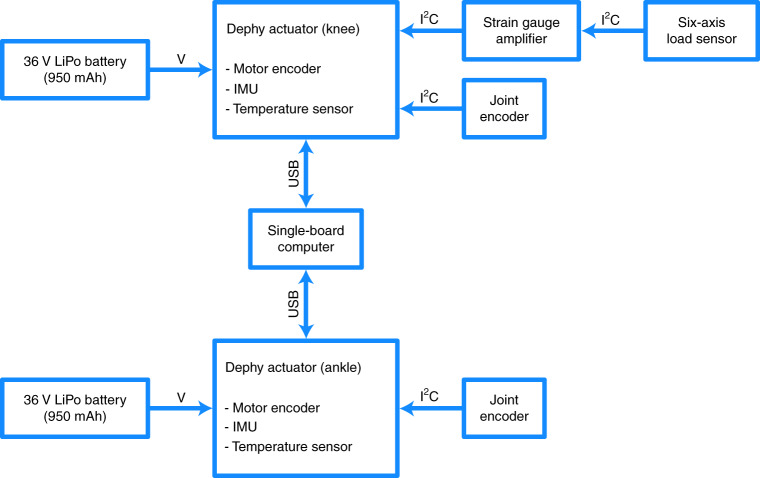
Recommended embedded system configuration. High-level overview of electronics, sensors and power supplies, along with the type of communication between components. The actuators are connected in parallel with a single-board computer (Raspberry Pi) by USB; this configuration was used for the benchtop testing, with a single motor. This is the recommended configuration because it does not require knowledge of specialized communication protocols; instead, the embedded system handles the inter-integrated circuit (I^2^C) and USB communications for the user. LiPo, lithium polymer; V, voltage.

Customization options enable the OSL to be suited to the individual uses of each researcher. The knee functions either as a series elastic actuator (SEA) or rigid actuator, and the stiffness of the series elasticity can be selected by the researcher using custom 100 N m rad^−1^ spring disks (Fig. 1). The springs fit inside the output pulley of the belt drive; the SEA configuration therefore does not change the volume of the OSL. Although the ankle does not include a specific series elastic element, it can integrate with either a compliant commercial foot or a rigid flat foot. The ankle has a maximum range of motion of 30°. In the default OSL configuration, this corresponds to 20° of plantarflexion (PF) and 10° of dorsiflexion (DF). By redesigning one component in the ankle, the amount of PF and DF can be modified within the 30° range; for example, we have also generated a version with 15° PF and 15° DF. The ability to change the operating region of the ankle provides researchers with additional customization based on their specific needs. In addition to hardware customization, researchers have multiple options for implementing high-level control. For example, we used two different embedded computers (Raspberry Pi 3 versus Texas Instruments DM3730) and communication protocols (USB versus serial peripheral interface) to control the motors in the benchtop and clinical tests (Fig. 2 and Supplementary Fig. 1). The OSL also functions with other high-level control schemes (such as MATLAB/Simulink, robot operating system) and external sensors (such as electromyography, additional IMUs).

### Benchtop testing

To facilitate the success of future researchers who may use the OSL, we completed electromechanical and thermal performance testing using a benchtop setup (Supplementary Videos 1 and 2). We tested low-level closed-loop position and current control, open-loop torque control and also tracked the OSL’s temperature increase during 70 min of continuous operation. Motor current is often used in prostheses to estimate output torque, and the performance of the current controller is critical for open-loop torque and impedance control. The series elasticity of the knee joint was not included in these tests—that is, the knee operated as a rigid actuator.

**Fig. 3:**
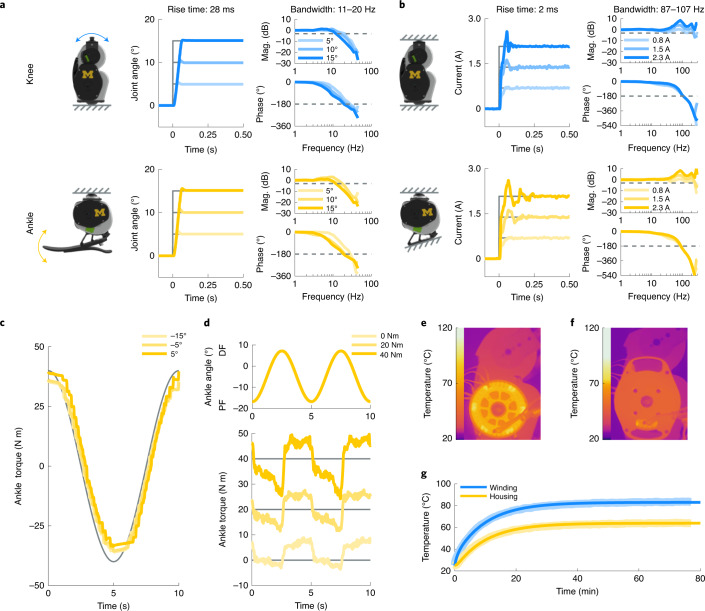
Electromechanical and thermal benchtop testing. **a**, The test setup, step response and frequency response for the closed-loop position control system. The output of each joint was free to rotate for these tests. The dashed lines represent the thresholds used to calculate the bandwidth. We tested each condition five times. Mag., magnitude. **b**, The test setup, step response and frequency response for the closed-loop current control system. The output of each joint was locked in place for these tests. We tested each condition five times. **c**, Open-loop torque tracking of a sinusoidal torque trajectory while the ankle prosthesis was mechanically grounded. We tested each condition three times. **d**, Open-loop torque tracking of a constant torque trajectory while the ankle prosthesis was sinusoidally rotated through its range of motion. We tested each condition three times. **e**, Representative thermal image of the knee prosthesis, without the embedded system mounting plate, after providing the motor with a constant current of 8 A for 70 min. The windings reach a steady-state temperature of 92 °C. **f**, Representative thermal image of the knee prosthesis, with the mounting plate, after providing the motor with a constant current of 8 A for 70 min. The windings reach a steady-state temperature of 83 °C. **g**, Simulated (bold) and experimental (shaded) thermal response of the motor to a constant current of 8 A. We tested each condition twice. Series elasticity was not included in these tests.

We characterized the performance of the two primary closed-loop controllers (position and current control) on the OSL. Step response tests were used to quantify the ability of the OSL to track changes in desired position and current reference values; frequency response tests estimated the bandwidth—that is, the range of input frequencies that the OSL can track with high fidelity—of the low-level position and current controllers (Supplementary Video 1). The position and current controllers exhibit fast and accurate step responses, with bandwidths of 10.7–20.2 Hz and 86.8–107.4 Hz, respectively (Fig. 3 and Table 1). Although we did not quantify torque bandwidth, we expect that it is much lower than the current control bandwidth, due to the dynamics of the transmission. Further results on the closed-loop controller performance can be found in our previous work^41^.

**Table 1:** OSL specifications compared with other prostheses and the human body

		OSL	MIT	Vanderbilt	Utah	Human^h^
Mass (g)	Knee	2,160–2,330^a^	2,700	2,700^e^	1,680	2,616^i^
	Ankle	1,740	2,000	2,300	1,045^f^	1,959^j^
Height (mm)	Knee	240	285	NA	290	314^i^
	Ankle	213	220	210	120	171^j^
Range of motion (°)	Knee	120	120	120	120	90
	Ankle	30	45	65	55	60
Transmission ratio	Knee	49	143	176	0–375	NA
	Ankle	58 ± 16^b^	170	116	50–800	NA
Series elasticity (N m rad^−1^)	Knee	~100–600	240	NA	NA	NA
	Ankle	NA^c^	1,688^e^	NA	NA	NA
Peak torque, continuous (N m)	Knee	50^d^	40	NA	39^e^	NA
	Ankle	59 ± 16^d^	NA	NA	NA	NA
Peak torque, instantaneous (N m)	Knee	150^c^	120	85	125	90
	Ankle	178 ± 49^d^	125	150	125	105
Peak speed (rad s^−1^)	Knee	5.2	NA	NA	NA	6.8
	Ankle	5.6	NA	NA	NA	5.4
Position bandwidth (Hz)	Knee	10–20	NA	NA	7^g^	2^k^
	Ankle	10–20	NA	NA	NA	4^k^
Torque constant (N m A^−1^)	Knee	0.14	0.028	0.028	0.014	NA
	Ankle	0.14	0.060	0.053	0.014	NA
Motor constant (N m W^−^^1/2^)	Knee	0.265	0.044	0.044	0.023	NA
	Ankle	0.265	0.057	0.096	0.043	NA
Bus voltage (V)	Knee	36	24	24	24	NA
	Ankle	36	24	24	24	NA

NA, not applicable/available.

^a^Knee mass varies with SEA configuration.

^b^The ankle transmission ratio profile is provided in Supplementary Fig. 7.

^c^The ankle does not have an explicit series elastic element; however, the carbon fibre foot provides some of the benefits of series elasticity without the added size, complexity or closed-loop torque control.

^d^Estimated with torque constant, transmission ratio, continuous (10 A) and instantaneous (30 A) motor current, and 90% efficiency at each transmission stage (73% overall efficiency).

^e^Estimated value.

^f^Does not include batteries or electronics.

^g^10° amplitude.

^h^Assuming a 75-kg, 1.7-m-tall participant walking on level ground or ascending/descending stairs.

^i^Assuming 75% shank mass and height.

^j^Assuming 25% shank plus foot mass and height.

^k^Defined as the frequency range over which 70% of the total signal power is captured.

Measuring torque is challenging in wearable robots because torque sensors with sufficient capacity can be large and heavy. Many groups instead use motor current as a substitute for output torque, resulting in an open-loop torque controller^20,42^. We quantified open-loop torque control performance using the ankle prosthesis—that is, these tests were accomplished without torque feedback. In the static condition, the ankle tracked a ±40 N m sinusoidal torque trajectory while locked in place (Fig. 3). In the dynamic condition, the ankle was sinusoidally rotated through most of its range of motion (16° PF to 8° DF) while tracking constant torques of 0 N m, 20 N m and 40 N m (Fig. 3). The root-mean-square error (r.m.s.e.) for torque tracking across the static and dynamic trials ranged from 4.4 N m to 7.4 N m.

The heat generated by an electric motor ultimately limits the torque it can generate, as well as how long it will operate safely. We therefore quantified the thermal response and developed a thermal model of the motor and OSL to a current step input of 8 A (Fig. 3 and Supplementary Video 2). Starting from an ambient temperature of 25 °C, the motor windings reached a steady-state temperature of 92 °C. After adding an additional housing (mounting plate for the embedded system), the windings and housing reached steady-state temperatures of 83 °C and 64 °C, respectively. That is, the embedded system, which behaves as a heatsink, improved the thermal response by 16%. Using these data, we developed a thermal simulation that predicts that the motor can operate at its continuous (10 A) and peak (30 A) current limits—corresponding to approximately 50 N m and 150 N m of joint torque (Table 1)—for 513 s and 17 s, respectively, before reaching potentially unsafe temperatures (set at 125 °C).

### Clinical testing

**Fig. 4:**
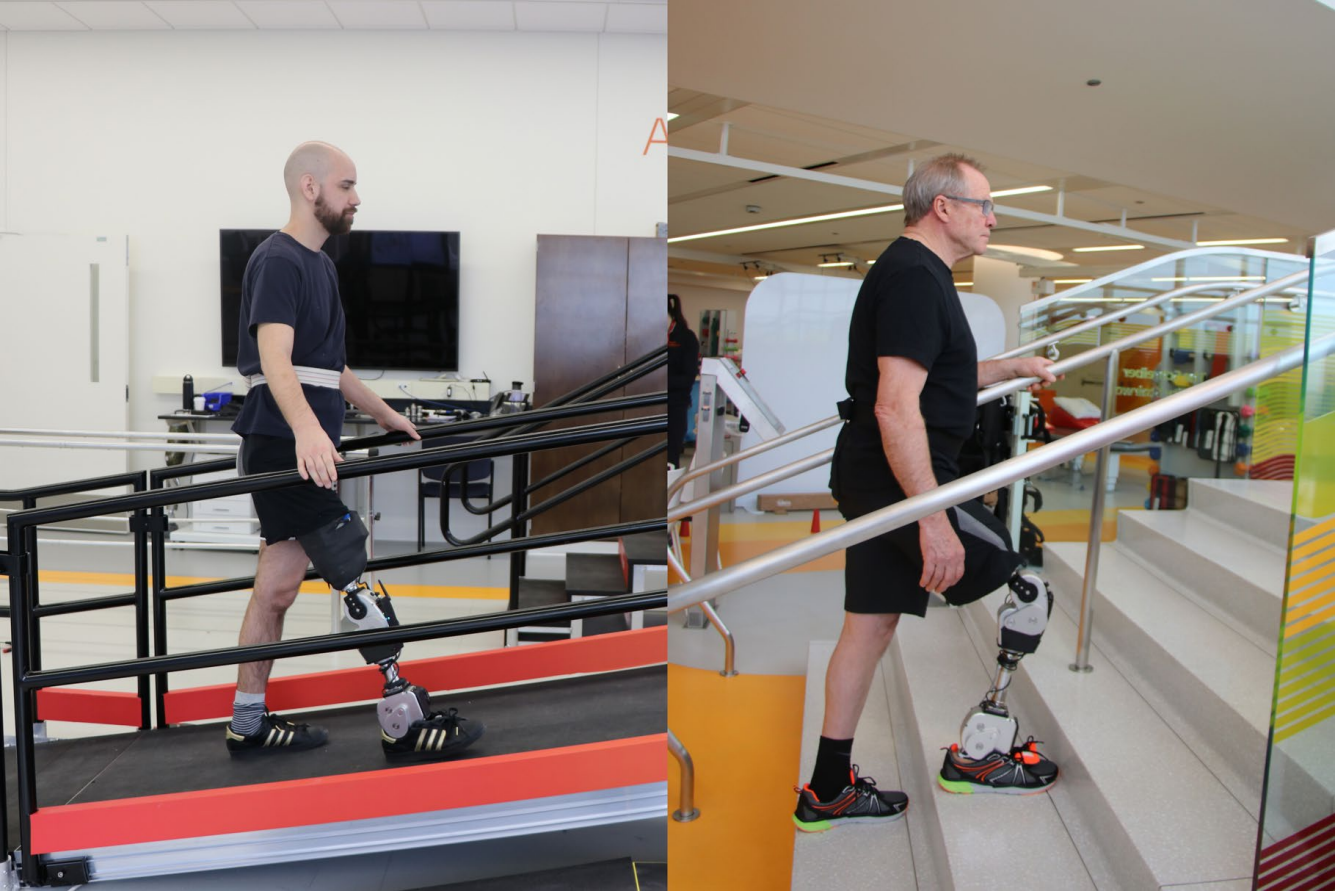
Participants with a transfemoral amputation ambulating with the OSL. Representative images of two participants ascending ramps and stairs throughout the hospital.

The OSL was tested clinically to highlight its kinematic and kinetic abilities in a real-world setting, demonstrate patients ambulating using the OSL, and provide biomechanical and control data for use as a benchmark for future researchers (Supplementary Video 1). We conducted clinical testing with three individuals with unilateral transfemoral (above-knee) amputations (Fig. 4 and Supplementary Table 1) who did not have previous experience with the OSL, but did have previous experience ambulating with other powered leg prostheses^20^. We implemented an impedance-based control system to enable different locomotion activities within a rehabilitation hospital^42^. Each participant provided written informed consent, approved by the Northwestern University Institutional Review Board.

**Fig. 5:**
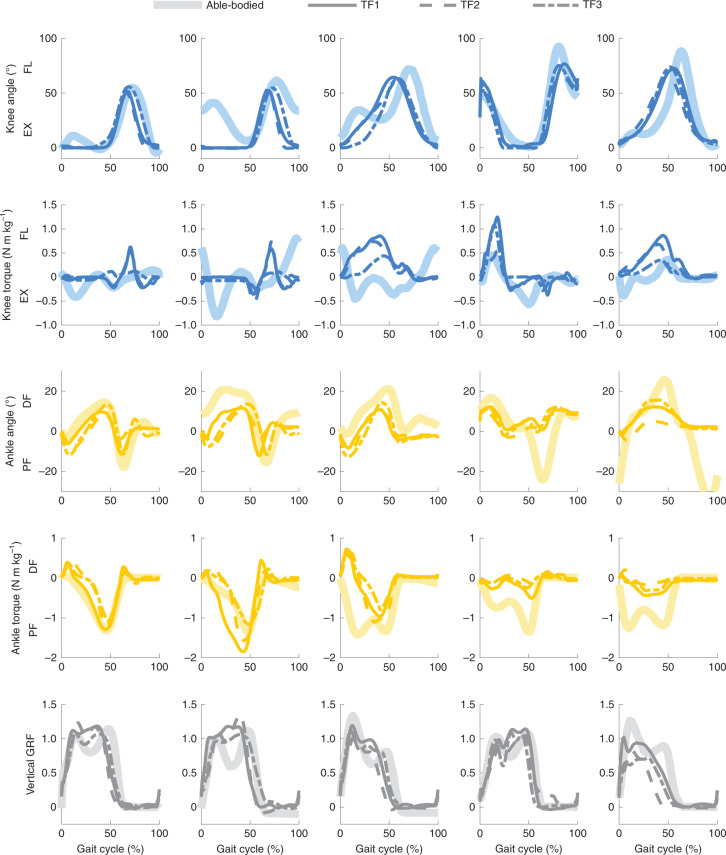
OSL kinematics and kinetics across five ambulation modes. Mean participant (TF) and able-bodied joint angles, moments and vertical GRFs. From left to right, walking, ramp ascent, ramp descent, stair ascent, stair descent. Joint torques are normalized by participant mass and GRF is denoted as a fraction of the participant weight. Knee flexion (FL) and extension (EX) as well as ankle DF and PF directions are included for clarity. Participants walked through the circuit 15–20 times (including training and tuning trials), resulting in approximately 380 analysed steps per participant for the walking condition and 38 analysed steps per participant for the ramp and stair conditions.

During ambulation, the OSL produced the desired joint torques (based on motor current); the r.m.s.e. between desired and actual current ranged from 0.5 A to 2.9 A per step, with peak currents ranging from 5 A to 25 A. The participants ambulated through a circuit that included level-ground walking, ramp ascent/descent and stair ascent/descent (Fig. 5). We quantified clinical success with a set of clinically relevant ambulation goals, developed by a physical therapist^42^; these goals were evaluated using the timing and magnitude of different kinematic and kinetic variables, along with visual inspection (Table 2). During level-ground and ramp ambulation, the OSL achieved heel strike with an extended knee (0.5 ± 0.7°, mean ± s.d.) and exhibited 7.4 ± 2.0° of controlled PF during early stance. The timings of push-off and knee extension were within 3.0 ± 2.7% and 6.0 ± 6.2% of able-bodied timing, respectively. Furthermore, the PF torque at push-off was 92.0 ± 23.4% of able-bodied torque. To enable swing clearance, the OSL produced 85.6 ± 7.8% of able-bodied knee flexion and 14.3 ± 10.1° of DF. Across all ambulation modes, the peak vertical ground reaction force (GRF) on the prosthetic side was 92.5 ± 17.2% of able-bodied GRF. Finally, participants ascended and descended stairs with a reciprocal gait pattern. Subjectively, participants noted during ambulation that the leg felt supportive, responsive and smooth.

**Table 2:** The clinical ambulation goals and performance metrics of the participants

Goal	Metric	Walking	Ramp ascent	Ramp descent	Stair ascent	Stair descent
Controlled weight acceptance	Knee extended at heel strike (°)	0.3 (−0.9)^a^	0.1 (33.7)	1.1 (8.9)	29.0 (50.1)	2.6 (2.3)
	Ankle early stance PF (°)	8.2 (3.8)	5.9 (0)	8.1 (3.3)	13.0 (15.2)	NA
Ambulation at desired speed	Ankle push-off time (%)	61.3 (64.0)	62.3 (66.0)	60.7 (66.0)	60.7 (64.0)	74.0 (74.0)
	Ankle push-off torque (N m kg^−1^)	1.2 (1.3)	1.5 (1.3)	1.0 (1.3)	0.3 (1.4)	0.3 (1.2)
	Knee swing extension time (%)	67.7 (72.0)	70.0 (77.0)	57.3 (69.0)	84.7 (81.0)	52.3 (63.0)
Appropriate amount of swing clearance	Knee swing flexion (°)	52.5 (55.0)	53.7 (61.7)	63.3 (71.8)	71.9 (92.5)	70.5 (88.3)
	Ankle swing DF (°)	12.0 (22.6)	12.6 (25.2)	1.9 (6.9)	10.1 (33.6)	NA
Minimal upper limb support	Peak vertical GRF (%)	116.4 (114.2)	119.7 (111.5)	111.5 (133.4)	109.8 (107.4)	85.4 (126.2)
Reciprocal gait pattern	Visual inspection	NA	Yes	Yes	Yes	Yes

NA, not applicable.

^a^Data of healthy individuals are shown in parentheses.

## Discussion

This study describes the design, implementation and characterization of an open-source robotic knee–ankle prosthesis, and demonstrates control with three participants with transfemoral amputations ambulating with the leg on level ground, ramps and stairs. The OSL is intended to be a simple, portable, scalable, customizable and economical hardware solution for the development and evaluation of control systems, both inside and outside of the laboratory (Fig. 1). Through the OSL, we hope to reduce the amount of time and resources that are needed to pursue prosthetics research, enable fair comparison between different control systems and provide long-term multi-study technology to accelerate progress in the field of powered prosthetic legs.

### Facilitating control research

To simplify usage and accelerate the adoption of the OSL, we developed a companion website (www.opensourceleg.com), which is a powerful resource that we hope will be a standard for open-source robotic hardware dissemination. The website includes solid model files, a bill of materials, links to suppliers, control system code, instructional guides, videos on assembly/disassembly and any other relevant information to improve the usability of the OSL (Supplementary Video 1). The content on the website provides the necessary documentation and videos to guide users through the steps required to (1) purchase the required machined and off-the-shelf components, (2) assemble/disassemble the OSL, (3) connect and communicate with external sensors, (4) perform benchtop controller experiments and (5) begin walking on level-ground with a simple impedance controller. We have also developed an online forum to allow researchers to post questions, results or independently developed modifications. Together, these tools will help researchers to use the OSL and encourage a more collaborative community focused on transforming the quality of life of individuals with amputations.

One of the greatest challenges to performing research with powered prostheses is developing reliable and robust mechatronic and embedded systems. We included embedded electronic systems that automatically implement sensing, motor commutation, low-level control, communication with other devices and safety protocols. By providing a built-in embedded system with the OSL, we are enabling researchers to focus on clinical testing and the development of higher-level control strategies instead of engineering and mechatronics of low-level embedded systems. As an open-source tool, the OSL also has the ability to integrate with other sensors and peripheral systems. For example, some users may be interested in adding electromyography (to measure muscle activity), force-sensitive resistors (pressure on the residual limb) or cameras (environment classification) to the OSL; others may be interested in providing real-time stimulation and sensory feedback directly to the user^38^. Although we do not currently use these systems with the OSL, they can be integrated quickly if they are compatible with Python or MATLAB, using the open-source embedded system—more-advanced systems may require further expertise. Researchers are encouraged to share their sensor systems and solutions on the OSL website to help to accelerate other groups.

Through this Article, we are also providing a baseline dataset for future controls research. This dataset includes prosthetic leg kinematics and kinetics for level-ground walking, ramp ascent/descent and stair ascent/descent, along with the impedance control parameters used. These data may serve as a starting point for researchers to test a clinically meaningful controller, or as a comparison point for control systems. Importantly, as this dataset encompasses multiple ambulation modes, it will enable testing in unstructured, uncontrolled environments much faster. These data will be hosted on the project’s website (www.opensourceleg.com).

### Design benchmarks and limitations

Prosthesis mass and size have a critical role in the success of these systems. Heavy prostheses require higher metabolic expenditure from the user^43^; this effect increases as the mass moves distally (that is, towards the ankle). Furthermore, as the build height of a prosthesis increases, fewer users can wear the leg—that is, if the prosthesis is too long, it will not fit below the residual limb. It is therefore critical to design lightweight and short prostheses. The OSL (~4,000 g) is lighter than most comparable prostheses (Table 1). The minimum build height of the OSL (~450 mm) is also comparable to the build height of other prostheses. Finally, the OSL is lighter and shorter than the foot and shank of a 75 kg adult with a height of 1.7 m (Table 1).

A limitation of the OSL is the range of motion of the ankle joint (Table 1). During most ambulation tasks, the biological ankle remains within 10° of DF and 20° of PF, as in the OSL; however, some individuals require a range of motion of 45–60° during stair descent^44,45^. Many other prosthetic ankles have a range of motion of 45–65°. The 30° range of motion of the OSL ankle is limited by the kinematics of the four-bar linkage, and could be improved by decreasing the ankle’s transmission ratio or using a different transmission design (Supplementary Fig. 2). However, individuals with amputations with passive prostheses can typically only achieve 10–15° during stair descent^46^. Therefore, although the OSL ankle does not achieve the full biological range of motion, it provides a substantial improvement over passive prostheses. The range of motion of the OSL knee is equal to other prostheses (120°), and is much higher than needed for typical ambulation tasks (70–90°; Table 1).

Transmission ratios determine prosthesis size, electrical demands, efficiency, performance and other factors. Using the high-torque drone motors, we reduced transmission ratios to 2–5 times lower than comparable prostheses (Table 1). The combination of high-torque motors and low transmission ratios enables the OSL to produce peak torques that are similar to other systems, while demonstrating higher bandwidth. For example, the OSL’s position bandwidth is approximately five times higher than the bandwidth of the biological knee or ankle; that is, the OSL is capable of recreating the human kinematics and kinetics (Table 1). The motors on the OSL have an overall winding-ambient thermal resistance of 3.9 K W^−1^, compared with the 7.6 K W^−1^ thermal resistance of motors used in other prostheses^14,20^. In addition to being 3–4 times more electrically efficient than these prostheses, the OSL’s motors produce 5–8 times less heat at steady state for a given joint torque (that is, after accounting for their respective transmission ratios; Supplementary Fig. 3).

The OSL is capable of locomotion for extended periods of time using the recommended batteries (36 V/950 mAh). During level-ground walking, the knee and ankle operate with an average power consumption of 9.3 W and 11.2 W, respectively, and the electronics operate at approximately 1–2 W. Given the energy in the batteries (34.2 W h), a user could walk continuously for approximately 2.8 h, 13.1 km or 8,750 strides (that is, 17,500 total steps) on a single charge, assuming consistent power consumption, a walking speed of 1.3 m s^−1^ and cadence of 104 steps per min^14^. The batteries lasted for the entire duration of our experiments (3 h). On average, individuals with lower-limb amputations walk approximately 6,000 steps per day, and healthy adults are considered to be active if they walk at least 10,000 steps each day^47,48^. The OSL batteries therefore have sufficient capacity for lab sessions and, potentially, daily ambulation.

### Clinical testing

**Fig. 6:**
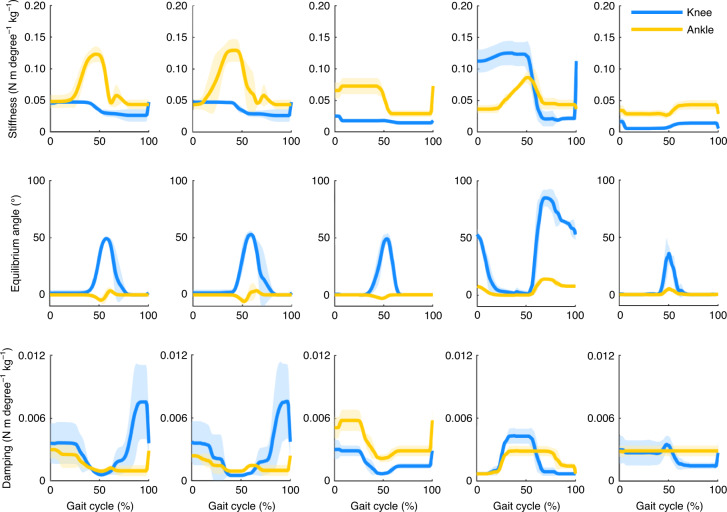
Tuned impedance parameters across five ambulation modes. Mean (bold) ± s.d. (shaded) of tuned stiffness, equilibrium angle and damping coefficient profiles for three participants. From left to right, walking, ramp ascent, ramp descent, stair ascent, stair descent.

All participants in this study ambulated on level ground, ramps and stairs using the OSL; all control parameter trajectories are provided for reference (Figs. 4–6). While walking on level ground and ramps, participants achieved PF during early stance, controlled DF during mid-stance and powered push-off during late stance. Participants descended ramps and stairs with a reciprocal gait pattern by taking advantage of stance-phase knee flexion; during stair ascent with a reciprocal gait, participants relied primarily on knee extension to propel themselves upwards and forwards.

Across most ambulation modes, joint angle and torque trajectories followed similar timing and amplitude patterns compared to data of healthy individuals^44,45,49^; however, there are notable differences. For example, participants did not demonstrate early-stance knee flexion during level ground walking (Fig. 5). At heel contact, patients with transfemoral amputations often pull back with their hip extensors to lock their prescribed, passive knee prosthesis into knee extension to prevent buckling and injury from falling; owing to this habit, the participants overrode the natural dynamics of the impedance controller in favour of an extended knee joint. Furthermore, participants relied primarily on the knee to ambulate up and down stairs; the ankle provided little torque and only rotated through approximately 30% of its range of motion.

It is important to note that the OSL is capable of producing early-stance knee flexion and ankle power across all ambulation modes; however, the participants exhibited compensatory movement based on their daily ambulation strategies with a passive prosthesis. We can overcome these compensatory motions through training (not shown), or by tuning the controller to recreate kinematics and kinetics of healthy individuals, but that was not the goal of this demonstration. We chose to not train the patients to overcome their compensatory strategies, because they needed to return to their daily-use prosthesis after the experiment—we had concerns that such training on the OSL might lead to a fall when they returned to their daily use device, which did require compensatory strategies. Instead, we tuned the OSL to meet a set of clinical ambulation goals (Table 2). Overall, our implementation succeeded in performing similarly to walking with the Vanderbilt Powered Leg using a similar impedance controller^42^.

### Outlook

We have described the design and mechanical, electrical and thermal evaluation of an open-source robotic knee–ankle prosthesis, and shown participants using the prosthesis across a range of activities, while providing controller parameters for reference. Future work includes advancement of the embedded systems, implementation of the SEA for closed-loop torque control and development of bioinspired impedance control policies^50,51^. We plan to produce an open-source version of our high-level controller that runs on a desktop or mobile device, providing researchers with a simple method of manually or automatically switching between ambulation modes and testing in non-steady-state conditions.

## Methods

### Design

#### Overview

The OSL incorporates a number of design innovations to facilitate adoption and improve performance. For example, we used a low-cost electric motor from the drone industry (Fig. 1 and Supplementary Table 2); as drone motors cannot rely on transmissions to increase torque output (due to the added mass), they produce 2–10 times more torque than the motors typically implemented in prosthetic legs. The motor’s increased torque density enabled us to implement transmission ratios of 2–5 times lower than comparable prostheses (Table 1). Transmissions amplify the torque produced by the motors, and higher ratios will require more stages; transmissions generally have an efficiency of 70–98% per stage, depending on transmission type^52^. Furthermore, the inertia reflected onto the motor increases with the square of transmission ratio. Thus, low transmission ratios are important in prostheses because they improve efficiency, electrical power demands, size and bandwidth, ultimately impacting mass, battery size, controllability and other factors^52,53^.

We designed the OSL through a multi-step design process that involved modelling human joint biomechanics, transmission kinematics, mechatronics (including motor electrical and thermal limitations) and the structural housing (Supplementary Fig. 4). This was an iterative design process with the overall goal of achieving able-bodied kinematics/kinetics while meeting the desired design principles, minimizing prosthesis mass/volume and satisfying mechatronic constraints.

#### Biomechanics

We began the design of the OSL by extracting the kinematic (for example, angle, angular velocity and angular acceleration) and kinetic (torque) trajectories of the knee and ankle joints from the literature. The data we used included walking at slow, self-selected and fast speeds, in addition to stair ascent/descent, for 75 kg and 100 kg participants^44^.

#### Transmission

We simulated the transmission and motor requirements for each of the knee and ankle prostheses by incorporating the governing equations of SEAs and d.c. motors, and using the extracted biomechanical joint angles (*θ*_l_) and torques (*τ*_l_) as the desired load requirements^14,54^. The motor mechanical requirements for a given ambulation mode are:

1 $$\theta _{\mathrm{m}} = \left( {\theta _{\mathrm{l}}} + \frac{\tau _{\mathrm{l}}}{k_{\mathrm{s}}} \right)N$$

2 $$\tau _{\mathrm{m}} = J_{\mathrm{m}}\ddot \theta _{\mathrm{m}} + \frac{{\tau _{\mathrm{l}}}}{{\eta N}}$$

where *θ*_m_, *τ*_m_ and *J*_m_ are motor angle, torque and inertia, respectively; *N* and *η* are transmission ratio and efficiency (assumed *η* = 0.9 in each stage), respectively; and *k*_s_ is the stiffness of the series elastic element. Using equations (1) and (2), we simulated a range of transmission ratios and stiffness values (including infinite stiffness for the rigid configuration) for each of the ambulation modes; these simulations helped to determine the range of acceptable transmission ratios and series elastic elements (Supplementary Fig. 5). On the basis of these simulations, we selected target transmission ratios of 49:1 and 40:1–100:1 for the knee and ankle, respectively.

Timing belts serve as the primary torque transmission mechanism in the OSL; specifically, we chose the recently developed PowerGrip GT3 (Stock Drive Products/Sterling Instrument) belts because they provide longer belt life, increased load-carrying capacity and quieter operation relative to other available belts^55^. Our overall goal when selecting belt/pulley configurations was to minimize the volume of each stage while resisting tooth jump. Tooth jump typically occurs with high torques or low belt tensioning, and is highly dependent on transmission geometry (that is, tooth profile, number of teeth of each pulley, number of teeth engaged, pulley centre-to-centre distance and belt width). However, there is little data available addressing the relationship between transmission geometry and the torque that endangers tooth jump. On the basis of the manufacturer’s documentation, we calculated conservative estimates of tooth jump torque (per mm belt width) for 2 mm, 3 mm and 5 mm pitch GT3 belts to be approximately 0.19 N m mm^−1^, 0.66 N m mm^−1^ and 2.4 N m mm^−1^, respectively^55^. We used these torque values to specify the minimum acceptable belt width for each transmission stage. To select the final belt drive geometries, we performed an iterative design process, investigating overall transmission volume, number of belt stages, pitch of each belt stage, width of each stage, number of teeth engaged, ease of assembly, and availability of belt lengths and widths. This process resulted in a 2 mm pitch stage followed by two 5 mm stages in the knee, and two 3 mm stages in the ankle (Supplementary Table 3). These belt drives were simulated to ensure that the torques at each stage would not lead to tooth jump during ambulation (Supplementary Fig. 6). The pulleys were machined from either 7075-T6 aluminium or 17-4 PH stainless steel using subtractive manufacturing, and the belts were purchased directly from the manufacturer.

In addition to a two-stage belt drive, the ankle prosthesis incorporates a four-bar linkage mechanism for torque transmission. We included the four-bar linkage instead of a third belt stage to reduce the overall size of the prosthesis and directly couple motion of the linkage to motion of the ankle joint and foot. To design the linkage, we began by simulating the range of motion and transmission ratio of more than 3,000 linkage configurations. The simulation varied the lengths of the individual links, through integer values between 1 and 10 units, and constrained the range of motion to a minimum of 20° angular distance between links to avoid singularities (Supplementary Fig. 2). Using these simulations, we explored a subset of linkage configurations while iterating through different belt drive options, and selected the mechanism that provided the best combination of range of motion, transmission ratio and size (Supplementary Fig. 2 and Supplementary Table 3). Increasing the range of motion of the linkage requires increasing the transmission ratio and size of the belt drive; we chose to limit the range of motion to 30° to reduce the overall size of the prosthesis. In contrast to the knee, which has a constant transmission ratio, the addition of the four-bar linkage resulted in a kinematically-varying transmission ratio for the ankle (Supplementary Fig. 7).

#### Series elasticity

The OSL also incorporates selectable series elasticity: the knee functions either as a SEA—and allows for modification of the series stiffness—or as a rigid actuator^56^ (Fig. 1). Selectable series elasticity enables researchers to use the SEA for energy storage/return, shock tolerance and torque control, whereas the rigid actuator is simpler, lighter and requires motor current to estimate torque. Depending on the desired magnitude of the series stiffness element, up to six torsional springs disks—each with a stiffness and mass of 97 ± 20 N m rad^−1^ and 23 g, respectively—can be stacked inside the transmission of the knee, resulting in a compact SEA without any added volume (Fig. 1).

Each spring disk has a thickness of 4.3 mm and contains 24 radially cantilevered beams, with a maximum deflection of 15°. At maximum deflection, the peak von Mises stress in the spring is approximately 250 MPa (half of 7075-T6 Aluminium’s yield strength). Torque measurement range increases with series stiffness; depending on the number of springs used, the measurable torque range is 25–150 N m. By contrast, torque measurement accuracy decreases with series stiffness; on the basis of the joint encoder’s resolution (0.02°), torque measurement resolution is 0.04–0.22 N m depending on the number of springs used.

#### Mechatronics

Given the mechanical requirements in equations (1) and (2), the brushed d.c. electromechanical model was used to determine the electrical demands:

3 $$i_{\mathrm{m}} = \frac{{\tau _{\mathrm{m}}}}{{k_{\mathrm{t}}}}$$

4 $$v_{\mathrm{m}} = i{_{\mathrm{m}}}R_{\mathrm{m}} + k{_{\mathrm{b}}}\dot \theta _{\mathrm{m}} + L_{\mathrm{m}}\frac{{{\mathrm{d}}i_{\mathrm{m}}}}{{{\mathrm{d}}t}}$$

where *i*_m_, *v*_m_, *R*_m_ and *L*_m_ are motor current, voltage, phase resistance and phase inductance, respectively; *k*_t_ is the torque constant; *k*_b_ is the back-emf constant (equivalent to the magnitude of *k*_t_ in SI units); and *t* is time. The current and voltage represent the *q*-axis current and voltage of field-oriented control, which enables a convenient analogue of the brushed electromechanical model. Using equations (3) and (4), we simulated various motor–battery combinations. Ultimately, the current and voltage demands (which are driven by the mechanical requirements) determined the overall mechanical design, power supply and motor selection.

We considered multiple motors for the OSL, including the 30 mm EC-4pole motor used in the MIT and Vanderbilt knee prostheses (305015, Maxon Motor) and a high-torque, exterior rotor motor (U8-16, T-motor) that has shown promising results in other areas of robotics^14,20,53,57–59^. The T-motor’s motor constant *k*_*m*_, which describes the motor’s ability to convert electrical energy to mechanical energy, is approximately 6 times higher than the EC-4pole’s motor constant (Table 1)—that is, the EC-4pole motor loses approximately 36 times more power than the T-motor for a given motor torque (Supplementary Fig. 3). However, as the EC-4pole motors are often coupled with larger transmission ratios, they do not have to produce the same amount of torque as the T-motor. We therefore simulated the T-motor with the transmission ratio of the OSL knee and the EC-4pole motor with the transmission ratios of the MIT and Vanderbilt knees to estimate the electrical power losses of the motors within a prosthesis. After accounting for transmission ratio, the EC-4pole motor loses approximately 3–4 times more power to heat for a given knee torque, when compared to the T-motor (Supplementary Fig. 3).

#### Housing

The OSL housing features a clamshell-style design, in which two halves are fastened together to enclose the prosthesis components (Fig. 1). The clamshell housings—which were machined from 7075-T6 Aluminium—have multiple purposes: they simplify the assembly process, reduce pinch points, locate the shafts for the timing belt pulleys and provide structural support for the OSL. The housings incorporate a system to properly apply tension to the belt stages; applying appropriate tension to the belt stages ensures that the transmission achieves maximum torque capacity and prevents tooth jump under load. The housings also include mechanical hard stops to ensure that the OSL remains within a biomechanically appropriate range of motion. All moving transmission components—except for the knee’s proximal pyramid and the ankle’s foot—are completely contained within the housing, preventing user injury and protecting the transmission from dirt and debris during testing in outdoor environments. The motors mount to the outside of the housings, enabling convenient assembly, removal and troubleshooting. Finally, the housings include space for batteries and electronics, creating a self-contained portable prosthesis.

#### Embedded system

The OSL’s embedded system integrates the motor with a commercial version of the Flexible Scalable Electronics Architecture—an open-source motor controller—in a compact, reliable package^60,61^ (Fig. 1). The embedded system implements low-level motor control and field-oriented control commutation; closes the feedback loops in the position, velocity, current and impedance controllers; and facilitates communication between the motor controller and external computers/sensors, using most common communication protocols.

To control overall prosthesis behaviour, researchers provide control commands (for example, desired position, desired current and controller gains) using their preferred hardware system (for example, microcontroller, laptop computer) and an open-source Python or MATLAB interface (Fig. 2). A graphical user interface is also available to quickly test the system, tune controllers, and display and save sensor data.

The OSL includes the following sensors: winding and bus electrical states, temperature, nine-axis IMU, a 14-bit motor encoder and a 14-bit joint encoder (Supplementary Table 2). An optional six-axis load cell can also be mounted to either the knee’s distal or the ankle’s proximal pyramid adaptor (Fig. 1). Finally, the embedded system includes a number of features to improve safety and reduce user error, including over- and under-voltage protection, over-current protection with programmable and physical fuses, and electrostatic discharge protection on the inputs and outputs.

### Benchtop testing

We performed several benchtop tests to quantify the OSL’s performance in both the time and frequency domains. We characterized closed-loop position and current controller performance, open-loop torque controller performance and tested the thermal response of the motor given a constant current input. In these tests and the paper as a whole, we report the *q*-axis current, which is analogous to the d.c. current in the standard brushed electromechanical model^62^.

A testing rig mechanically grounded the knee and ankle joints, and provided a reaction torque, during the current control tests; during the position control tests, the joints were free to rotate (Supplementary Fig. 8). For these benchtop tests, we used a single-board computer (Supplementary Table 2) to send the desired current and position trajectories (through USB at approximately 750 Hz) to the Dephy actuator, which subsequently performed low-level feedback control (Fig. 2). We tested the knee and ankle separately, and did not test with series elasticity.

We quantified the OSL’s ability to track desired position and current commands by conducting step response tests (Fig. 3). We commanded motor encoder steps corresponding to 5°, 10° and 15° steps at the joint, starting from the midpoint of the range of motion; we also commanded current steps of 2 A, 4 A and 6 A (peak-phase current), corresponding to 0.8 A, 1.5 A and 2.3 A of *q*-axis current, respectively. We performed five trials for each condition.

To quantify the range of frequencies in which the OSL can track position and current commands, we performed frequency response tests (Fig. 3). The position trajectories were Gaussian white noise signals—third order, 40 Hz low-pass filtered—scaled to ±5°, ±10° and ±15° amplitudes, and centred at the midpoint of the range of motion. The current trajectories were also Gaussian white noise signals—third order, 200 Hz low-pass filtered—scaled to ±0.8 A, ±1.5 A and ±2.3 A amplitudes, and centred at 0 A. The position trials lasted for 15 s, whereas the current trials lasted for 60 s. We conducted five trials for each condition and constructed Bode plots using Blackman–Tukey spectral analysis, in which the auto-spectrum and cross-spectrum are divided in the frequency domain^63^. Using the Bode plots, we calculated the bandwidth as the frequency in which the magnitude crossed −3 dB.

We estimated the ankle’s open-loop torque controller in both static and dynamic conditions by rigidly mounting the ankle prosthesis to the Neurobionics Lab Rotary Dynamometer (Supplementary Fig. 8). The dynamometer includes a frame-mounted motor (BSM90N-3150AF, Baldor) and a six-axis load cell (45E15A4, JR3). In the static condition, the dynamometer locked the ankle at angles of −15°, −5° and 5°, where negative values correspond to PF and positive values correspond to DF. The ankle prosthesis, without torque feedback, tracked a ±40 N m, 0.1 Hz sinusoidal torque trajectory. In the dynamic condition, the dynamometer rotated the ankle through a 24°, 0.2 Hz sinusoidal angle trajectory; while being rotated, the ankle prosthesis, without torque feedback, tracked constant torque values of between 0 N m and 40 N m. Each trial lasted 10 s, and we conducted three trials in each condition.

To test the thermal behaviour of the OSL we supplied the motor with a constant current of 8 A (6.5 A *q*-axis current) across two winding leads for 70 min using a power supply (1688B, B&K Precision), and measured the resulting change in winding and housing temperature using a thermal imaging camera (ONE Pro LT, FLIR Systems). The T-motor is delta wound, which means that, when current is supplied between two leads, one phase has twice the current of the other two phases (Fig. 3). As a consequence, we estimated winding temperature to be the weighted average of the more and less powered windings. We subsequently modelled the thermal dynamics of the motor by simulating the equivalent electrical circuit (Supplementary Fig. 9) in Simulink^22,64^. In this model, temperature and heat flow are analogous to voltage and current, respectively; heat flow is equivalent to the thermal power lost (*P*_loss_) through the electrical resistance of the motor, which is a function of temperature:

5 $$P_{{\mathrm{loss}}} = i_{\mathrm{m}}^2R_{\mathrm{m}} = i_{\mathrm{m}}^2R_{{\mathrm{m}},\,{\mathrm{A}}}(1 + \alpha _{{\mathrm{Cu}}}(T_{\mathrm{w}} - T_{\mathrm{A}}))$$

where *R*_m,A_ is the motor electrical resistance at ambient (room) temperature, *α*_Cu_ is copper’s temperature coefficient of resistance, and *T*_w_ and *T*_A_ are winding and ambient temperature, respectively. We used the measured temperature data to calculate the motor’s optimal thermal parameters: winding-housing thermal resistance and capacitance (*R*_th,w_ and *C*_th,w_), as well as housing-ambient thermal resistance and capacitance (*R*_th,h_ and *C*_th,h_).

Although we did not use series elasticity in the benchtop experiments and the experiments in individuals with amputations, we did characterize the stiffness of the elastic elements when included inside the knee output pulley (Fig. 1). We mounted the knee onto a six-axis load sensor (45E15A4, JR3) and manually rotated the knee from 0° to 15° and back. We performed five trials each for the following configurations: 0, 1, 2, 3, 4, 5 and 6 springs. We also locked the input shaft to ensure that the knee’s rotation was due to spring deflection instead of belt drive motion.

### Clinical testing

To test the OSL’s ability to restore gait and demonstrate real-time impedance control on the hardware, we used a previously developed control approach^42^. We implemented locomotion controllers for standing, level-ground walking, ramp ascent/descent and stair ascent/descent using impedance control; the impedance parameters for each ambulation mode regulated the current to the knee and ankle motors on the basis of the desired torque^65^. The desired motor current was determined by converting the desired joint torque *τ*_j_, into motor current:

6 $$\tau _{\mathrm{j}} = - k_{\mathrm{j}}\left( {\theta _{\mathrm{j}} - \theta _{0{\mathrm{j}}}} \right) - b_{\mathrm{j}}\dot \theta _{\mathrm{j}}$$

where j corresponds to the knee or ankle joint, *θ*_j_ is joint angle (positive values represent knee extension and ankle DF) and $\dot \theta _{\mathrm{j}}$ is joint angular velocity. The three tunable impedance parameters for each joint were virtual stiffness *k*_j_, virtual equilibrium angle *θ*_0j_ and virtual damping coefficient *b*_j_ (Fig. 6). The desired joint torque was converted to the desired motor torque using the transmission ratio, and the desired motor current was calculated using equation (3).

Within our tuning process, a finite-state machine divided all gait activities (except for standing) into four subphases: early-to-mid stance, late stance, swing flexion and swing extension; simple logic on the basis of mechanical sensors within the prosthesis (for example, joint encoders and load sensor) enabled progression through the state machine. The standing mode controller used only two states—the first was relatively stiff to support the weight of the body when the prosthesis was in contact with the ground and the second enabled the leg to swing freely when it was not in contact with the ground.

For 60% of the states (across all ambulation modes), we held impedance parameters at tuned but constant values. For the remaining 40% of the states, we modulated the impedance parameters according to one of the following control laws: (1) basing impedance parameters on values from the previous state; (2) mimicking biological joint responses (that is, modifying joint impedance as a function of ankle angle); (3) modifying joint impedance as a function of knee angle; or allowing users to control the rate of power generation/dissipation—that is, modifying joint impedance as a function of (4) decreasing, or (5) increasing prosthesis load. We discussed each of these approaches in detail previously^42^. These control strategies were used to reduce the number of independent parameters that are required to tune the prosthesis and improve transitions between different types of activities. This control scheme created an overall system response that enabled each participant to walk safely, comfortably and confidently. For these clinical tests, we used an embedded microcontroller (Supplementary Table 2) to perform high-level control (at approximately 40 Hz) and send the desired current trajectories to the embedded system, which subsequently performed low-level feedback control; the microcontroller and embedded system communicated by serial peripheral interface (Supplementary Fig. 1).

During the first visit, a certified prosthetist fitted and aligned the OSL to each participant, ensuring suspension, comfort and stability on the leg during standing. Participants next walked within a set of parallel bars while we tuned the impedance parameters for level-ground walking. Participants subsequently ambulated up/down stairs and up/down ramps while we tuned the impedance parameters using a combination of visual inspection and feedback from the prosthetist, a physical therapist and the participant (Fig. 6). Tuning continued until a set of clinical ambulation goals—including appropriate weight acceptance, PF, knee power, swing clearance, step length, walking speed and minimal upper extremity support (Table 2)—were met, and the prosthetist, therapist and participants were satisfied with the OSL’s performance^42^. This visit lasted approximately 2–3 h.

During the second visit, participants performed a series of ambulation circuits that included all of the following activities: standing, walking, stair ascent, stair descent, ramp ascent and ramp descent, as described previously^39,66^. The circuit included seamless transitions between activities, achieved using a mobile phone that communicated with the embedded controller^67^. Seamless transitions within the experiment included the following: standing to walking, walking to standing, walking/standing to stairs, stairs to walking/standing, walking to ramps and ramps to walking. We instructed participants to ambulate at a comfortable speed and recorded data using the OSL’s on-board sensors. This visit also lasted approximately 2–3 h.

### Reporting summary

Further information on research design is available in the Nature Research Reporting Summary linked to this article.

## Supplementary information

Supplementary Information Supplementary figures and tables and captions for Supplementary Videos 1 and 2.

Reporting Summary

Supplementary Video 1 Assembly, testing and ambulation.

Supplementary Video 2 Thermal response to a constant current input of 8 A across two winding leads.

## Data Availability

The data supporting the results in this study are available within the paper and its Supplementary Information, and at the project website (www.opensourceleg.com).

## Code availability

Solid model files, a list of materials, links to suppliers, control system code, instructional guides and videos on assembly/disassembly are available at the project website (www.opensourceleg.com).
