# Supplementary material for: Design and clinical implementation of an open-source bionic leg
Source: Nat Biomed Eng. 2020 Oct 5;4(10):941–53. doi: 10.1038/s41551-020-00619-3 (PMC7581510; doi:10.1038/s41551-020-00619-3)
Supplement: Supplementary file 1 — Supplementary figures and tables and captions for Supplementary Videos 1 and 2. [file 41551_2020_619_MOESM1_ESM.pdf]

natureresearch
https://doi.org/10.1038/s41551-020-00619-3

Supplementary information

Design and clinical implementation of an open-source bionic leg

In the format provided by the authors and unedited

Contents

Fig. S1 | Clinical testing embedded system configuration....2
Fig. S2 | Simulated range of motion and transmission ratio for various linkage configurations....2
Fig. S3 | Motor and prosthesis efficiency comparison....3
Fig. S4 | Overview of the OSL design process....4
Fig. S5 | Simulated motor speed-torque curves for the knee and ankle prostheses....4
Fig. S6 | Simulated belt drive torques for the knee and ankle prostheses....5
Fig. S7 | Ankle transmission ratio throughout the range of motion....5
Fig. S8 | Benchtop testing setup....6
Fig. S9 | Equivalent electrical circuit used to model the thermal dynamics of the motor....7
Table S1 | Subject characteristics....8
Table S2 | Hardware model information....8
Table S3 | Transmission stage specifications....8
Movie S1 | OSL assembly, testing, and ambulation....9
Movie S2 | Thermal response to a constant current input of 8 A across two winding leads....9

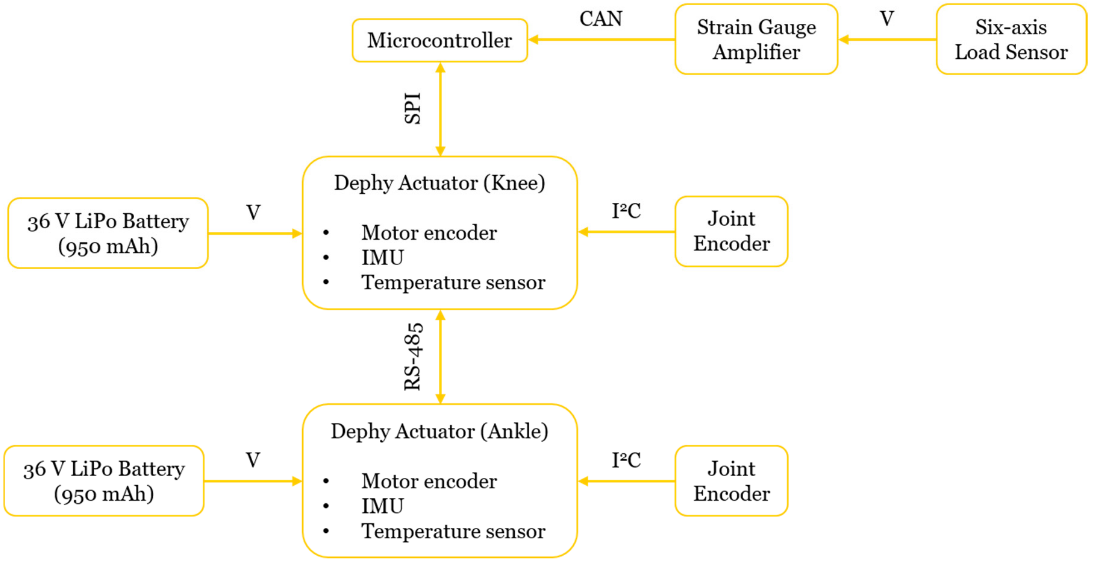

Fig. S1 | Clinical testing embedded system configuration. The actuators are connected in series with a microcontroller, and the load sensor communicates with the microcontroller via Controller Area Network (CAN) bus; this configuration was used for the clinical testing.

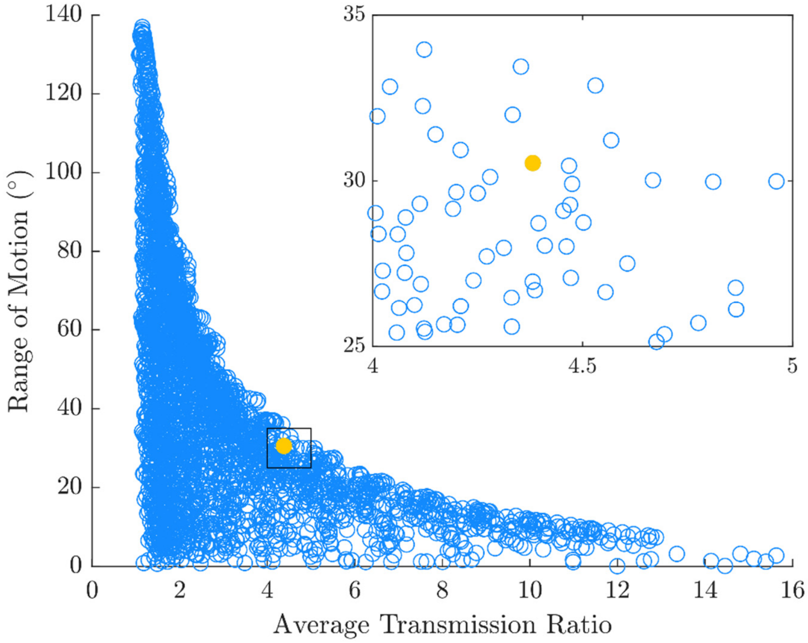

Fig. S2 | Simulated range of motion and transmission ratio for various linkage configurations. Design of the linkage required a tradeoff between ankle range of motion and transmission ratio. Filled yellow circle denotes the linkage implemented in the ankle prosthesis. The inset shows a magnified view of the linkage options similar to the selected linkage.

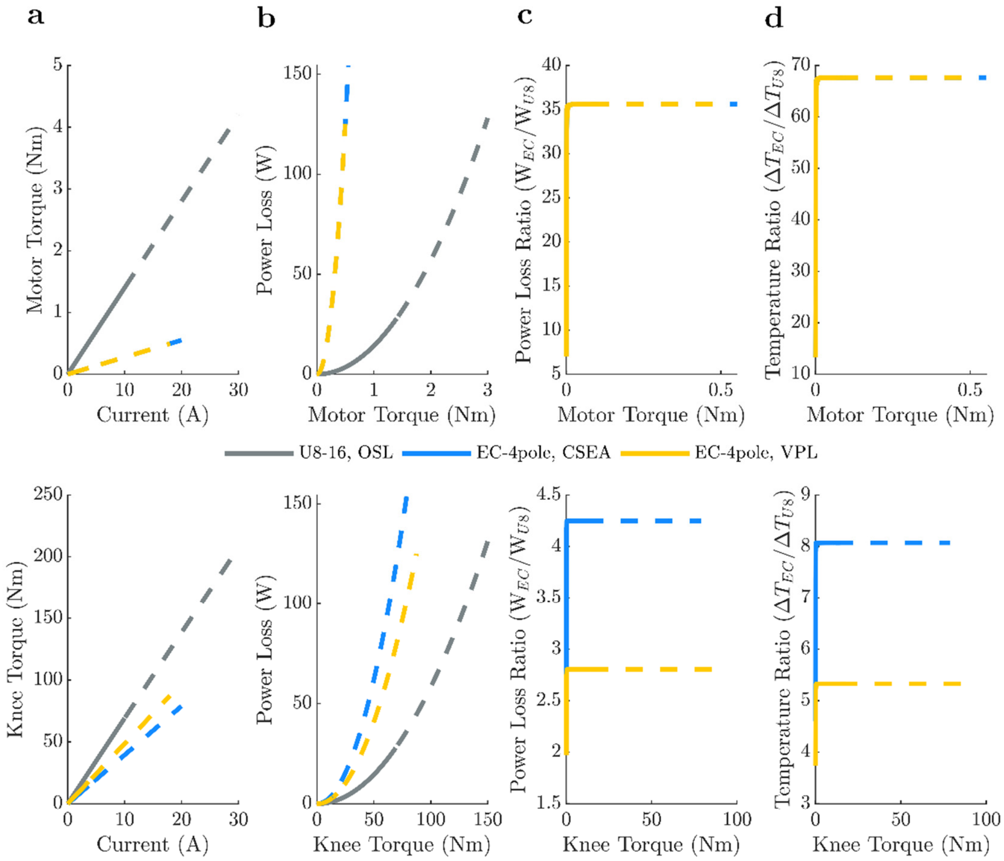

Fig. S3 | Motor and prosthesis efficiency comparison. Simulated comparison of the (top) T-motor and Maxon motor used in the MIT Clutchable Series-Elastic Actuator (CSEA) and Vanderbilt Powered Leg (VPL), and of the (bottom) motors coupled to the transmission ratio used in their respective prostheses[14,20]. **a**, Torque-current relationship in the continuous (solid) and peak (dashed) current regions. **b**, Electrical power loss (i^2R) as a function of torque, assuming zero angular velocity. **c**, Ratio of the EC-4pole motor's power loss to the U8-16 motor's power loss. **d**, Ratio of the EC-4pole motor's temperature increase to the U8-16 motor's temperature increase.

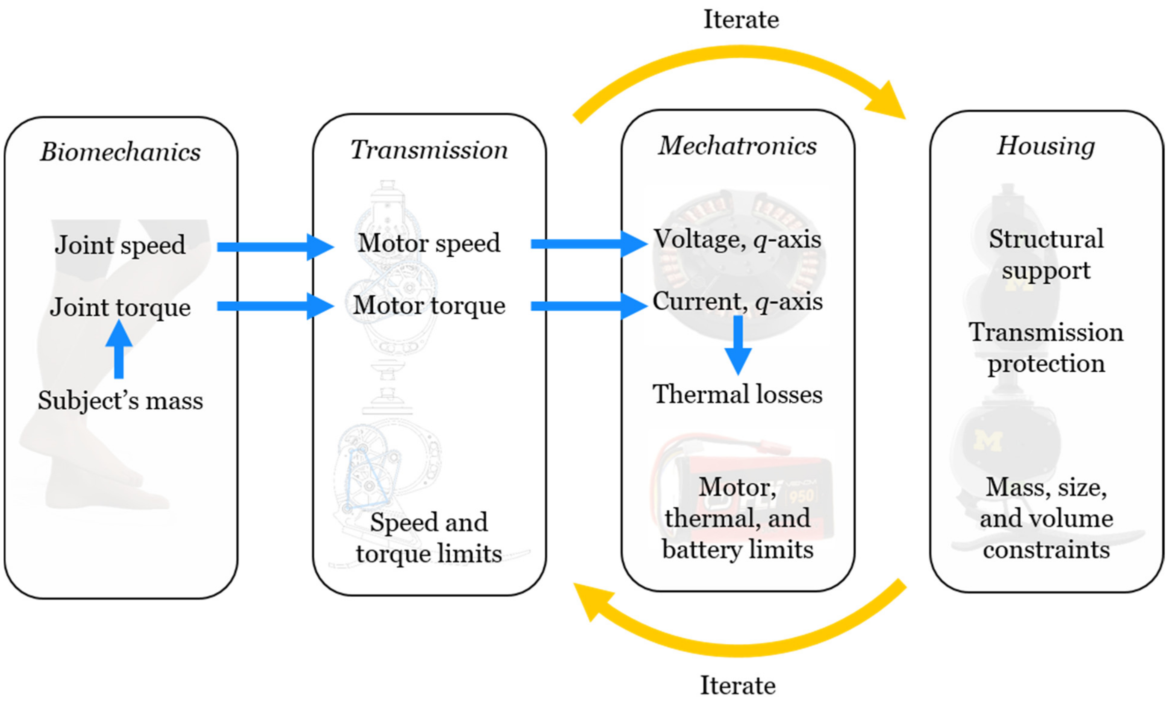

Fig. S4 | Overview of the OSL design process. Able-bodied kinematics and kinetics provide load requirements, which are transmitted into motor kinematic (voltage) and kinetic (current) requirements. The final transmission, battery, and housing designs resulted from iterating to meet speed, torque, electrical, thermal, mass, and size constraints.

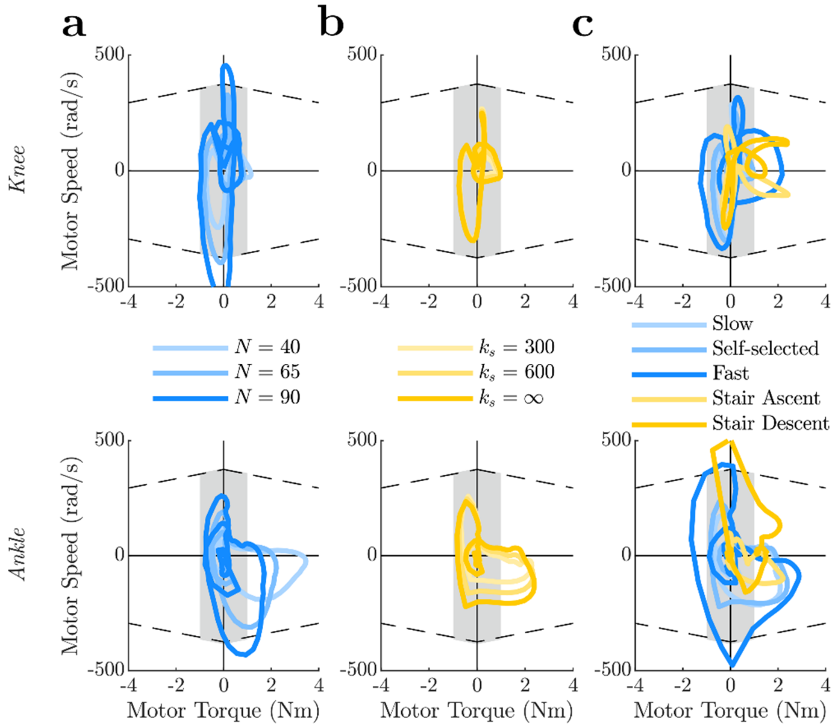

Fig. S5 | Simulated motor speed-torque curves for the knee and ankle prostheses. a, Transmission ratio simulation in a rigid configuration for self-selected walking. **b**, SEA simulation with the OSL transmission ratios for self-selected walking. **c**, Ambulation mode simulation in a rigid configuration using the OSL transmission ratios. Shaded area denotes the continuous operating region.

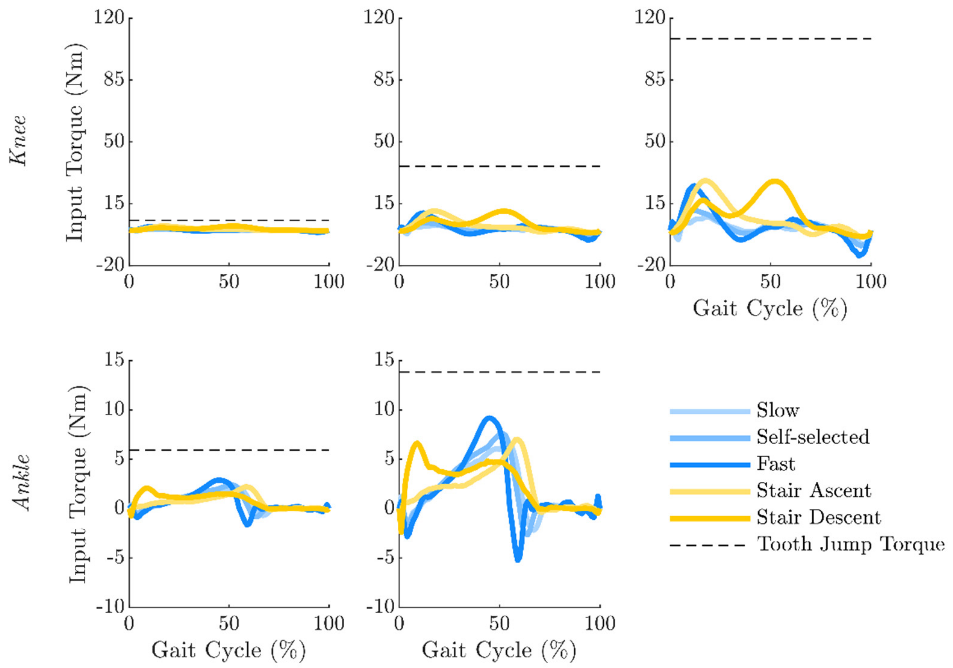

Fig. S6 | Simulated belt drive torques for the knee and ankle prostheses. The expected torques applied to the input pulleys of the belt drive stages are below the tooth jump torque across all the simulated ambulation modes. From left to right: Stage 1, Stage 2, and (knee only) Stage 3.

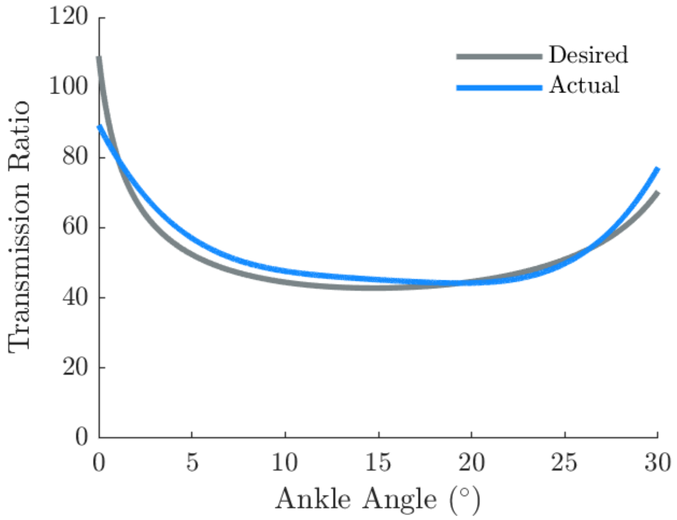

Fig. S7 | Ankle transmission ratio throughout the range of motion.

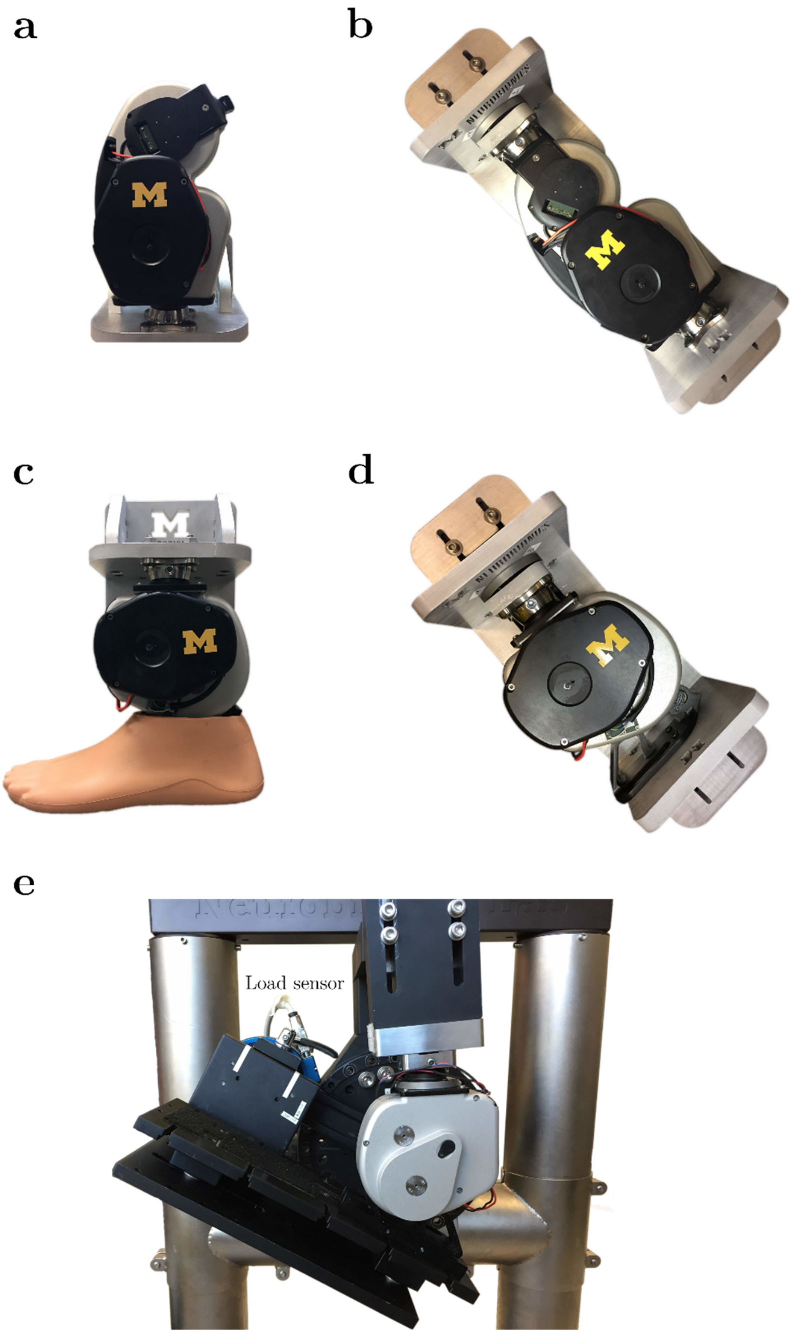

Fig. S8 | Benchtop testing setup. a, Knee closed-loop position controller testing—knee joint is free to rotate. **b**, Knee closed-loop current controller and thermal response testing. **c**, Ankle closed-loop position controller testing—ankle joint is free to rotate. **d**, Ankle closed-loop current controller testing. **e,** Ankle open-loop torque controller testing. The dynamometer either locked (static trials) or rotated (dynamic trials) the ankle joint while the prosthesis commanded open-loop torque values. The external load sensor was used to measure output torque.

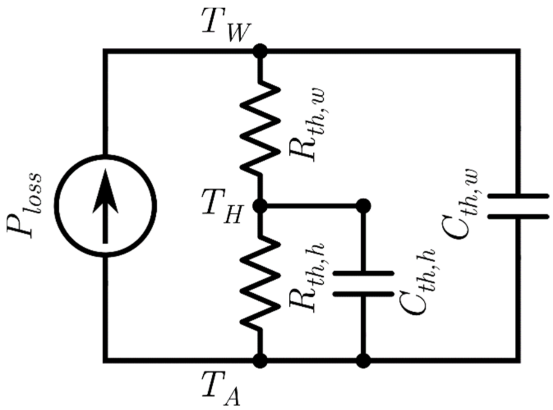

Fig. S9 | Equivalent electrical circuit used to model the thermal dynamics of the motor. We used the measured temperature data to calculate the motor's thermal resistances and capacitances.

Table S1 | Subject characteristics.

Subject	Gender	Age (years)	Time Post-amputation (years)	Etiology	Weight (kg)	Height (m)	Mobility Level	Prescribed Knee Prosthesis
TF1	F	51	29	Traumatic	61.7	1.65	K3	Rheo
TF2	M	33	2	Sarcoma	63.1	1.77	K3	Genium
TF3	M	70	44	Traumatic	86.2	1.75	K3	C-Leg

Table S2 | Hardware model information.

Item	Model	Company	Location
Motor	U8-16	T-motor	Nanchang, Jiangxi, China
Batteries	25087X2	Venom Power	Rathdrum, ID, USA
Temperature Sensor	MCP9700A	Microchip Technology Inc.	Chandler, AZ, USA
IMU	MPU-9250	InvenSense	San Jose, CA, USA
Motor Encoder	AS5047P	ams AG	Premstaetten, Austria
Joint Encoder	AK7452	Asahi Kasei Microdevices Corporation	Tokyo, Japan
Load Sensor	M3564F	Sunrise Instruments	Nanning, China
Single-board Computer	Raspberry Pi 3	Raspberry Pi Foundation	Cambridge, UK
Embedded Microcontroller	DM3730	Texas Instruments	Dallas, TX, USA

Table S3 | Transmission stage specifications.

		Knee			Ankle		
		Stage 1	Stage 2	Stage 3	Stage 1	Stage 2	Stage 3
Belt Drive	Pitch (mm)	2	5	5	3	3	-
	Input Pulley Teeth	16	14	14	17	17	-
	Output Pulley Teeth	80	40	48	60	60	-
	Transmission Ratio	5.00	2.86	3.43	3.53	3.53	-
	Belt Teeth	92	50	60	72	72	-
	Belt Width (mm)	30	15	45	9	21	-
Linkage	Crank Length (mm)	-	-	-	-	-	2
	Coupler Length (mm)	-	-	-	-	-	10
	Rocker Length (mm)	-	-	-	-	-	7
	Frame Length (mm)	-	-	-	-	-	10
	Transmission Ratio	-	-	-	-	-	3.44-8.75
	Range of Motion (°)	-	-	-	-	-	30.54

Movie S1 | OSL assembly, testing, and ambulation.

Movie S2 | Thermal response to a constant current input of 8 A across two winding leads. We tested each condition 2 times.
