# Supplementary material for: Design and clinical implementation of an open-source bionic leg
Source: Nat Biomed Eng. 2020 Oct 5;4(10):941–53. doi: 10.1038/s41551-020-00619-3 (PMC7581510; doi:10.1038/s41551-020-00619-3)
Supplement: Supplementary file 2 — Reporting Summary [file 41551_2020_619_MOESM2_ESM.pdf]

nature research

Corresponding author(s): Elliott J. Rouse
Last updated by author(s): Jul 31, 2020

Reporting Summary

Nature Research wishes to improve the reproducibility of the work that we publish. This form provides structure for consistency and transparency in reporting. For further information on Nature Research policies, see Authors & Referees and the Editorial Policy Checklist.

Statistics

For all statistical analyses, confirm that the following items are present in the figure legend, table legend, main text, or Methods section.

n/a	Confirmed	
☐	☒	The exact sample size (*n*) for each experimental group/condition, given as a discrete number and unit of measurement
☒	☐	A statement on whether measurements were taken from distinct samples or whether the same sample was measured repeatedly
☒	☐	The statistical test(s) used AND whether they are one- or two-sided<br>*Only common tests should be described solely by name; describe more complex techniques in the Methods section.*
☒	☐	A description of all covariates tested
☒	☐	A description of any assumptions or corrections, such as tests of normality and adjustment for multiple comparisons
☐	☒	A full description of the statistical parameters including central tendency (e.g. means) or other basic estimates (e.g. regression coefficient) AND variation (e.g. standard deviation) or associated estimates of uncertainty (e.g. confidence intervals)
☒	☐	For null hypothesis testing, the test statistic (e.g. *F*, *t*, *r*) with confidence intervals, effect sizes, degrees of freedom and *P* value noted<br>*Give P values as exact values whenever suitable.*
☒	☐	For Bayesian analysis, information on the choice of priors and Markov chain Monte Carlo settings
☒	☐	For hierarchical and complex designs, identification of the appropriate level for tests and full reporting of outcomes
☒	☐	Estimates of effect sizes (e.g. Cohen's *d*, Pearson's *r*), indicating how they were calculated

Our web collection on statistics for biologists contains articles on many of the points above.

Software and code

Policy information about availability of computer code

Data collection	Data from the benchtop testing were collected using a Raspberry Pi and Python 3. Data from the clinical testing were collected using a Texas Instruments DM3730 microcontroller and C/C++.
Data analysis	All data were analyzed using MATLAB R2018a.

For manuscripts utilizing custom algorithms or software that are central to the research but not yet described in published literature, software must be made available to editors/reviewers. We strongly encourage code deposition in a community repository (e.g. GitHub). See the Nature Research guidelines for submitting code & software for further information.

Data

Policy information about availability of data

All manuscripts must include a data availability statement. This statement should provide the following information, where applicable:

- Accession codes, unique identifiers, or web links for publicly available datasets
- A list of figures that have associated raw data
- A description of any restrictions on data availability

The data supporting the results in this study are available within the paper and its Supplementary Information, and on the project website (www.opensourceleg.com).

Field-specific reporting

Please select the one below that is the best fit for your research. If you are not sure, read the appropriate sections before making your selection.

☒ Life sciences ☐ Behavioural & social sciences ☐ Ecological, evolutionary & environmental sciences

For a reference copy of the document with all sections, see nature.com/documents/nr-reporting-summary-flat.pdf

Life sciences study design

All studies must disclose on these points even when the disclosure is negative.

Sample size	No sample-size calculation was performed as this was a first-in-human technology demonstration, and no statistical analyses were performed with regard to the subject data. We felt that three subjects of varying age and background were sufficient to demonstrate that the technology could be fit and tuned to multiple individuals.
Data exclusions	No data were excluded from the analyses.
Replication	Each subject could reliably ambulate with the device on two separate sessions. Thus, replication of the technology was demonstrated by having three different subjects ambulate using the device on two separate occasions.
Randomization	Subjects only ambulated with a single experimental device. Consequently, no randomization was performed.
Blinding	Blinding was not possible during this experiment because the subject could see the physical device and feel the mass of the device (which was different than their clinically prescribed prosthesis) during ambulation.

Reporting for specific materials, systems and methods

We require information from authors about some types of materials, experimental systems and methods used in many studies. Here, indicate whether each material, system or method listed is relevant to your study. If you are not sure if a list item applies to your research, read the appropriate section before selecting a response.

Materials & experimental systems

n/a	Involved in the study	
☒	☐	Antibodies
☒	☐	Eukaryotic cell lines
☒	☐	Palaeontology
☒	☐	Animals and other organisms
☐	☒	Human research participants
☒	☐	Clinical data

Methods

n/a	Involved in the study	
☒	☐	ChIP-seq
☒	☐	Flow cytometry
☒	☐	MRI-based neuroimaging

Human research participants

Policy information about studies involving human research participants

Population characteristics	Three subjects (1 female; age 33–70 years; mass 61.7–86.2 kg) with unilateral above-knee amputation.
Recruitment	The Shirley Ryan AbilityLab maintains an amputee registry: a list of individuals who have an amputation and are interested in being contacted about potential research studies. Individuals from the registry were contacted after the study was authorized by the Northwestern University Institutional Review Board (IRB). Recruitment materials were not in any way coercive, and subjects were free to decline study participation at all stages. Each subject was initially approached by his/her own personal physician, prosthetist, or occupational therapist, so initial discussions were private and protected. Informed consent to participate in the study was then obtained per IRB protocol. There was no restriction on the inclusion of women or minorities; however pregnant women were excluded from the study, in case of a fall.<br><br>These subjects were chosen to participate in this study as they are K3-K4 ambulators. They are able to ambulate at various speeds, walk without assistive devices, tolerate walking on various terrain, and maintain single-limb balance if needed. The three subjects in this dataset were a convenience sample and chosen due to their availability to come into the research hospital for in-lab testing. Likely a different set of K3/K4 level ambulators would still show near-similar kinematics and the ability to walk on the OSL as long as a prosthetist can suspend a heavier device than their daily use prosthesis without compromising residual-limb skin health. The time needed for proper alignment, configuration of the prosthesis parameters, and practice using the device may vary between subjects, but our previous experience teaching individuals with a transfemoral amputation how to use a powered leg prosthesis demonstrates that nearly all ambulators at this level can learn how to use the powered prosthesis for level walking, incline walking and stair climbing.

Ethics oversight	Each subject provided written informed consent, approved by the Northwestern University Institutional Review Board.

Note that full information on the approval of the study protocol must also be provided in the manuscript.
